# Accelerated triacylglycerol production without growth inhibition by overexpression of a glycerol-3-phosphate acyltransferase in the unicellular red alga *Cyanidioschyzon merolae*

**DOI:** 10.1038/s41598-018-30809-8

**Published:** 2018-08-17

**Authors:** Satoshi Fukuda, Eri Hirasawa, Tokiaki Takemura, Sota Takahashi, Kaumeel Chokshi, Imran Pancha, Kan Tanaka, Sousuke Imamura

**Affiliations:** 1 School of Life Science and Technology, Tokyo Institute of Technology, Yokohama, Japan; 2 Interdisciplinary Graduate School of Science and Engineering, Tokyo Institute of Technology, Yokohama, Japan; 3 Laboratory for Chemistry and Life Science, Institute of Innovative Research, Tokyo Institute of Technology, Yokohama, Japan

## Abstract

Microalgae accumulate triacylglycerols (TAGs), a promising feedstock for biodiesel production, under unfavorable environmental or stress conditions for their growth. Our previous analyses revealed that only transcripts of *CmGPAT1* and *CmGPAT2*, both encoding glycerol-3-phosphate acyltransferase, were increased among fatty acid and TAG synthesis genes under TAG accumulation conditions in the red alga *Cyanidioschyzon merolae*. In this study, to investigate the role of these proteins in TAG accumulation in *C. merolae*, we constructed FLAG-fused CmGPAT1 and CmGPAT2 overexpression strains. We found that CmGPAT1 overexpression resulted in marked accumulation of TAG even under normal growth conditions, with the maximum TAG productivity increased 56.1-fold compared with the control strain, without a negative impact on algal growth. The relative fatty acid composition of 18:2 in the TAGs and the *sn*−1/*sn*−3 positions were significantly increased compared with the control strain, suggesting that CmGPAT1 had a substrate preference for 18:2. Immunoblot analysis after cell fractionation and immunostaining analysis demonstrated that CmGPAT1 localizes in the endoplasmic reticulum (ER). These results indicate that the reaction catalyzed by the ER-localized CmGPAT1 is a rate-limiting step for TAG synthesis in *C. merolae*, and would be a potential target for improvement of TAG productivity in microalgae.

## Introduction

Microalgae produce biomass using sunlight, water and carbon dioxide by the process of photosynthesis. The biomass production rate of many microalgal species generally exceeds that of land plants^1,2^. Microalgae store energy in storage molecules, such as neutral lipids that form cytoplasmic lipid droplets (LDs). The accumulated lipid is mainly in the form of triacylglycerols (TAGs), which can be used for biodiesel production^1^. In contrast to corn-based ethanol or soy- and palm-based biodiesel, biofuels derived from microalgal feedstocks can be produced free of competition with resources used for agricultural food production^3^. Moreover, microalgae can grow under severe environmental conditions and thus can be cultivated with many types of culture systems on an industrial scale^1,3,4^. Due to these reasons, microalgae have attracted considerable interest as a promising biofuel resource.

A few key genes for TAG accumulation have been identified, mostly in the unicellular green alga *Chlamydomonas reinhardtii*. Boyle *et al*.^5^ identified a regulatory gene, *NRR1*, encoding a SQUAMOSA promoter-binding protein domain transcription factor, which was induced during nitrogen depletion. NRR1 is considered an important regulator of TAG synthesis under nitrogen depletion because the *nrr1* mutant produces only ~50% TAG compared with the wild-type^5^. PDAT1, phospholipid:diacylglycerol acyltransferase 1, contributes to TAG accumulation under nitrogen-depletion conditions because mutants obtained by insertional mutagenesis or artificial microRNA silencing for *PDAT1* show reduced TAG contents^5,6^. It has also been shown that galactoglycerolipid lipase (PGD1) is required for TAG accumulation under nitrogen-depletion conditions^7^. PGD1 hydrolyzes monogalactosyl-diacylglycerol into its lyso-lipid derivative, and galactoglycerolipid is a source of fatty acids for TAG synthesis. With respect to improving TAG production, it was reported that TAG production was increased by overexpression of *CrDGTT4*, encoding a type-2 diacylglycerol acyltransferase (DGAT), under phosphorus-depletion conditions in *C. reinhardtii*^8^. Recently, Ajjawi *et al*.^9^ reported that lipid production in *Nannochloropsis gaditana* was doubled under nitrogen-replete conditions by decreasing the expression of a homolog of a fungal Zn(II)_2_Cys_6_, which determines total carbon partitioning. However, concurrent microalgal cell growth and high-yield TAG production under normal growth conditions, which is most important for improving TAG productivity, has been achieved only in a few cases^10^.

*Cyanidioschyzon merolae* is a unicellular red alga that lives in acid hot springs (pH 1–3, 40–50 °C), with each cell containing only one mitochondrion, one chloroplast, and one nucleus. The complete genome sequences of these three organelles were determined^11^, and it was revealed that they have simple, minimally redundant gene contents. Because these biological characteristics have been elucidated and various tools have been established for analysis of *C. merolae*^12–14^, this alga is considered a good model organism to understand various molecular functions of photosynthetic eukaryotes, including the fundamental regulation of TAG production. In previous studies, we constructed rapamycin-sensitive *C. merolae* strains by expressing the *Saccharomyces cerevisiae* FKBP12 protein in the cells^15,16^. Using the resultant strains, we revealed that inactivation of target of rapamycin (TOR), a conserved serine/threonine protein kinase that plays a central role in the regulation of a cell growth and metabolism^17–20^, by rapamycin leads to accumulation of LDs and TAGs in the cells. TAG accumulation has been observed not only in *Chlamydomonas reinhardtii* but also in *Arabidopsis thaliana* by TOR-inactivation with TOR-specific inhibitors^21–24^, indicating TOR is a checkpoint kinase for TAG accumulation in divergent plant lineages. However, how TOR controls TAG synthesis is still unknown. In this study, we investigated the role of the endoplasmic reticulum (ER)-localized glycerol-3-phosphate acyltransferases (GPATs), CmGPAT1 and CmGPAT2, in TAG accumulation in *C. merolae*, and found that the reaction catalyzed by the ER-localized CmGPAT1 is a rate-limiting step for TAG synthesis in this alga. We suggest that ER-localized GPATs are potential targets for increasing the TAG productivity in microalgae.

## Results

### CmGPAT1 and CmGPAT2 are endoplasmic reticulum-localized GPATs in *C. merolae*

**Figure 1:**
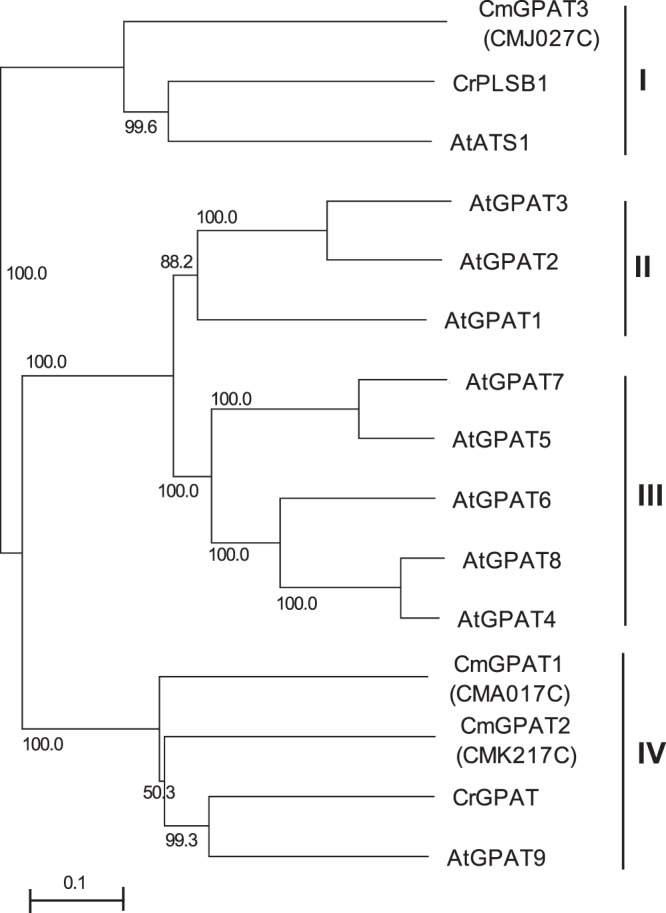
Phylogenetic tree of algal and plant GPATs. A neighbor joining tree based on 303 unambiguously aligned amino acid proteins was constructed. Numbers at each node represent percentages from 1,000 bootstrap replications. Branch lengths are proportional to the number of amino acid substitutions, as indicated by the scale bar below the tree. Designations and GenBank accession numbers for the sequences of each protein are as follows: CmGPAT1, CmGPAT2, and CmGPAT3 for *Cyanidioschyzon merolae* CMA017C, CMK217C, and CMJ027C (http://merolae.biol.s.u-tokyo.ac.jp/); AtGPAT1, AtGPAT2, AtGPAT3, AtGPAT4, AtGPAT5, AtGPAT6, AtGPAT7, AtGPAT8, AtGPAT9, and AtATS1 for *A. thaliana* GPATs (NP_563768.1, NP_563651.1, NP_001329010.1, NP_171667.1, NP_187750.1, NP_181346.1, NP_196227.1, NP_191950.2, NP_568925.1, and NP_174499.1); and CrGPAT and CrPLSB1 for *Chlamydomonas reinhardtii* GPATs (AFC93411.1 and XP_001694977.1). I, II, III, and IV denote groups defined in this study.

Our previous transcriptomics analysis revealed that only transcripts of *CMA017C* (hereafter *CmGPAT1*) and *CMK217C* (*CmGPAT2*), both encoding glycerol-3-phosphate acyltransferase (GPAT), were upregulated among fatty acid- and TAG-synthesis genes under TAG-accumulation conditions, nitrogen depletion and TOR-inactivation (Fig. S1)^22^. This finding raised the possibility that either or both of these genes play an important role in TAG accumulation in *C. merolae*. Before performing a detailed analysis to check this possibility, we first performed a phylogenetic analysis to determine whether the two proteins might be GPATs responsible for TAG biosynthesis because plant GPATs have diverse functions^25^. As shown in Fig. 1, the GPAT proteins used in the analysis were divided into four groups, I–IV. CmGPAT1 and CmGPAT2 were categorized into group IV, which contains *Chlamydomonas reinhardtii* GPAT (CrGPAT) and *Arabidopsis thaliana* GPAT9 (AtGPAT9). Previous studies revealed that CrGPAT was localized in oil bodies^26^ and AtGPAT9 is an endoplasmic reticulum (ER)-localized GPAT responsible for lipid and TAG biosynthesis^25,27,28^. The *C. merolae* nuclear genome encodes another GPAT, CMJ027C (CmGPAT3), which is classified into group I with the chloroplast-localized *A. thaliana* ATS1 (AtATS1) and *C. reinhardtii* PLSB1 (CrPLSB1)^29^. It has been reported that AtGPAT1*–*8 (groups II and III) are land plant-specific GPATs involved in cutin and suberin synthesis^30–33^. Thus, the phylogenetic tree analysis suggested that CmGPAT1 and CmGPAT2 are ER-localized GPATs involved in TAG synthesis.

**Figure 2:**
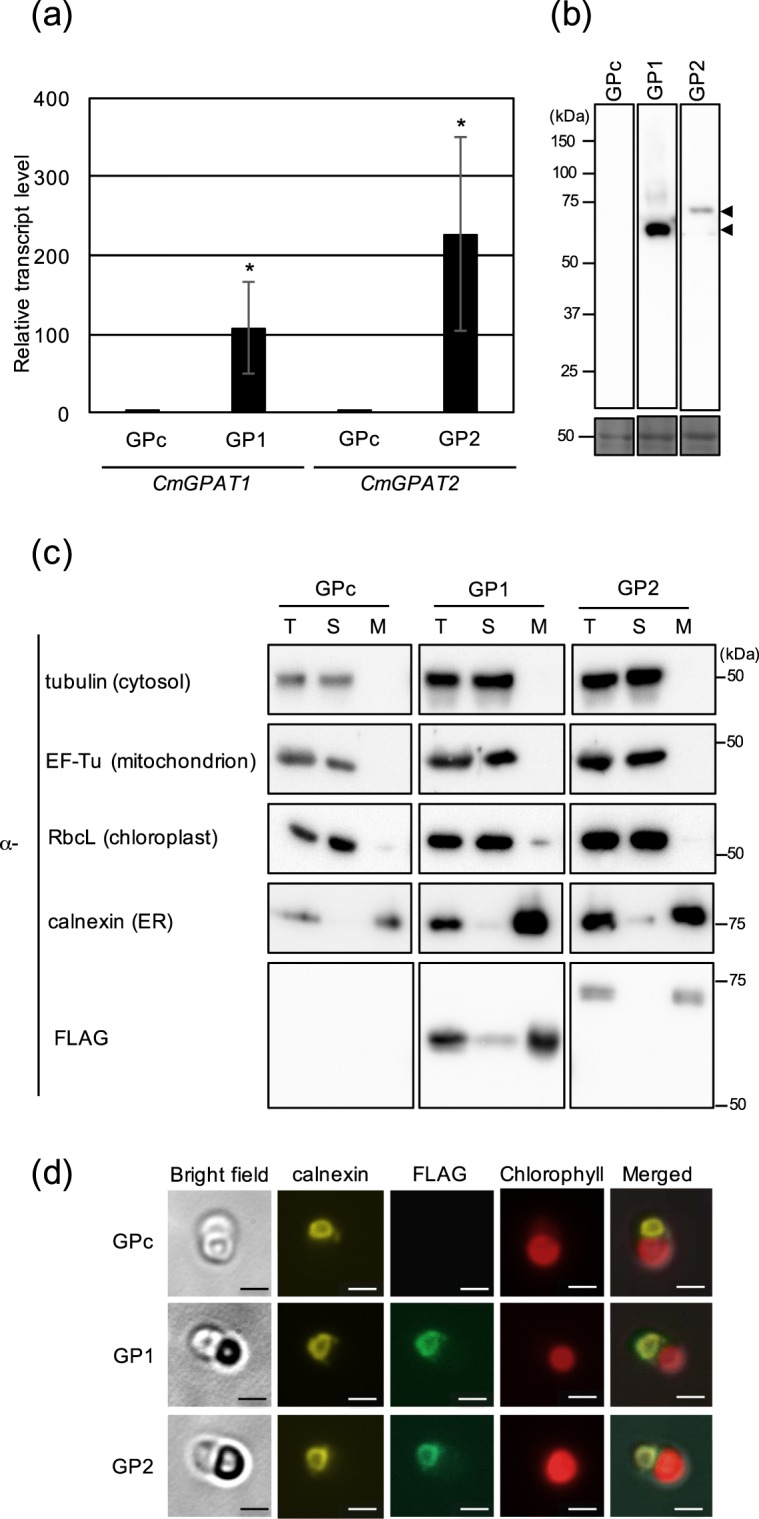
Overexpression of CmGPAT1 and CmGPAT2 and their intracellular localization. (**a**) Transcript levels of *C. merolae CmGPAT1* and *CmGPAT2* genes in each overexpression strain. The levels of *CmGPAT1* and *CmGPAT2* transcripts in the GP1 and GP2 strains were analyzed by quantitative real-time PCR and are presented as relative values (mean of *n* = 3 ± S.D.; the value for the control strain GPc is normalized to 1.0). (**b**) Expression of FLAG-fused CmGPAT1 and CmGPAT2. Aliquots of total protein (6 µg each) isolated from the indicated strains were separated by 10% sodium dodecyl sulfate-polyacrylamide gel electrophoresis (SDS-PAGE) and analyzed by immunoblotting with a monoclonal anti-FLAG antibody. Molecular size marker positions are indicated in kDa on the left. The arrowheads indicate the expected positions of the FLAG-tagged CmGPAT1 and CmGPAT2 proteins. After antibody detection of the signal, the membrane was stained with Coomassie Brilliant Blue, which was used as a loading control (lower panel). (**c**) Immunoblot analysis of FLAG-fused CmGPAT1 and CmGPAT2 after cell fractionation. Total protein (T), soluble (S), and microsome (M) fractions were isolated from GPc, GP1, and GP2, and subjected to immunoblot analysis with the indicated antibodies. Tubulin, EF-Tu, RbcL, and calnexin were used as marker proteins for the cytosol, mitochondrion, chloroplast, and endoplasmic reticulum (ER), respectively. Molecular size marker positions are indicated in kDa on the right. (**d**) Intracellular localization of FLAG-fused CmGPAT1 and CmGPAT2. Fluorescence derived from calnexin (yellow signal, calnexin), FLAG-fused CmGPAT1 or CmGPAT2 (yellow-green signal, FLAG), intrinsic chlorophyll fluorescence (red signal, Chlorophyll), merged image of calnexin, FLAG, and Chlorophyll (Merged), and bright field image (Bright field) in indicated strains are shown. Bars correspond to 1 μm.

To further examine their intracellular localization and roles in TAG synthesis in the cell, we constructed CmGPAT1 and CmGPAT2 overexpression strains, named GP1 and GP2, respectively. The GPc strain, into which the empty plasmid vector was introduced, was used as the control. Quantitative real-time PCR (qRT-PCR) analysis revealed that transcripts of *CmGPAT1* and *CmGPAT2* were increased approximately 110- and 230-fold in GP1 and GP2, respectively, compared with GPc (Fig. 2a). Furthermore, FLAG-fused CmGPAT1 and CmGPAT2 proteins were detected at their predicted molecular weights in the relevant strains in immunoblot analysis, indicating that the CmGPAT1 and CmGPAT2 overexpression strains were successfully constructed (Fig. 2b).

Using the FLAG epitope tag as a marker, we investigated the intracellular localization of CmGPAT1 and CmGPAT2 in samples after cell fractionation (Fig. 2c). Given that both GPATs are localized in the ER, the proteins should be detected in microsomes, which are artificial structures derived from pieces of ER. FLAG-fused CmGPAT1 and CmGPAT2 were abundantly detected in microsomal fractions, and the band patterns were similar to those of ER-localized calnexin^34^. Bands corresponding to cytosolic tubulin^34^, mitochondrion-localized EF-Tu^35^, and chloroplast-localized RbcL^36^ were observed in the soluble fraction, but not in the microsomal one. We further confirmed the intracellular localization of CmGPAT1 and CmGPAT2 by indirect immuno-fluorescence microscopy analysis. Yellow-green fluorescence showing the CmGPAT1 and CmGPAT2 signal was observed from a limited area in the cytosol, and the signal co-localized with a yellow fluorescence derived from calnexin (Fig. 2d). These phylogenetic, immunoblot, and indirect immuno-fluorescence microscopy analyses clearly indicated that both CmGPAT1 and CmGPAT2 are ER-localized GPATs in *C. merolae*.

### Accumulation of LDs and TAG by overexpression of CmGPAT1 under normal growth conditions

**Figure 3:**
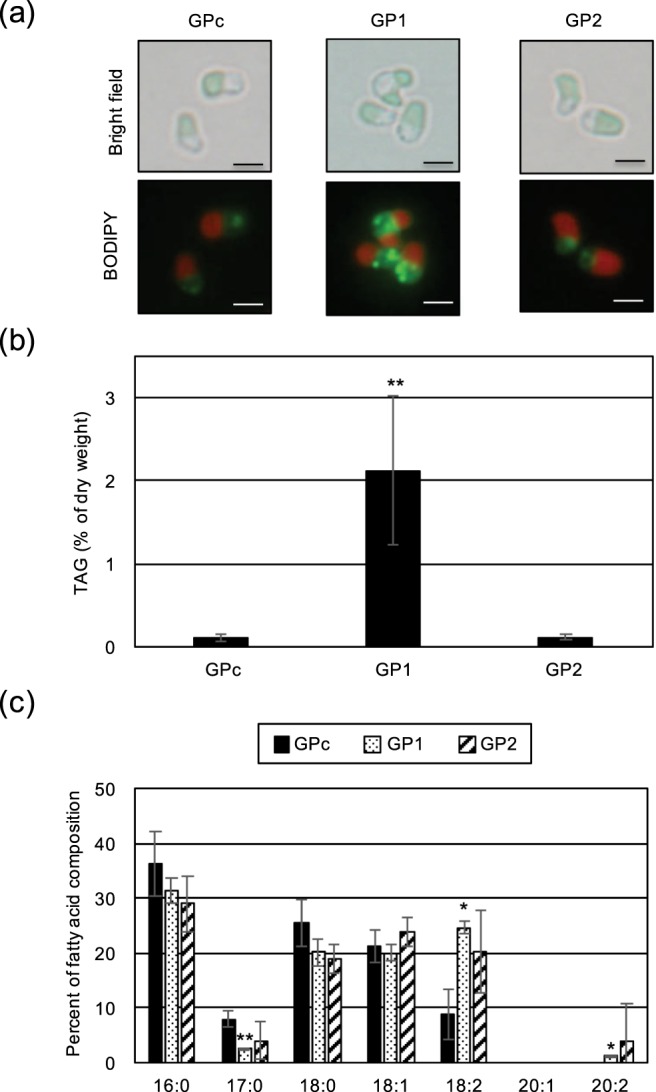
Accumulation of lipid droplets and TAGs by CmGPAT1 overexpression. (**a**) BODIPY staining of GPc, GP1, and GP2 cells. Each cell was grown under normal growth conditions until OD_750 = _0.4–0.6, and was stained with BODIPY. Bright field (top) and BODIPY staining (bottom) images are indicated. Each BODIPY staining (yellow-green signal) image was merged with the relevant chlorophyll fluorescence (red signal) image. *Bar*, 2 μm. (**b**) Intracellular TAG content in GPc, GP1, and GP2 cells. Values are averages of three independent experiments and represent percentages of dry weight. Error bars indicate standard deviation (SD). Asterisks indicate a significant difference compared with GPc (Student’s *t*-test, *P* < 0.05). (**c**) Fatty acid composition of the purified TAGs in GPc, GP1, and GP2 cells. Fatty acid components of the TAGs are indicated as a percentage of the fatty acid composition. Each fatty acid is indicated by the number of carbons:number of double bonds. Values are averages of three independent experiments. *Error bars* indicate SD. Asterisks indicate a significant difference compared with GPc (Student’s *t*-test, *P < 0.05; **P < 0.01).

If the ER-localized CmGPAT1 and CmGPAT2 play critical roles in TAG synthesis in *C. merolae*, overexpression of each GPAT may lead to accumulation of cytoplasmic LDs containing TAG. We thus first examined whether LD formation was induced in the GP1 and GP2 strains under normal growth conditions. In BODIPY staining analyses, obvious LD formation was observed in GP1, but not in the GP2 or GPc strains (Fig. 3a). Consistent with the LD accumulation, the TAG content in GP1 was significantly increased by approximately 19-fold compared with the levels in GPc (Fig. 3b). As shown in Fig. 3c, the relative cellular contents of 17:0 (number of carbons:number of double bonds) and 18:2/20:2 fatty acids in the TAGs in GP1 were decreased and increased, respectively, in comparison with those in GPc. However, the TAG content and TAG fatty acid composition in GP2 were not significantly changed in comparison with those in GPc. In both overexpression strains, the morphology, cell size, and total lipids extracted from each cell were not significantly changed in comparison with the control strain (Figs 3a and S2). These results clearly suggested that a step catalyzed by CmGPAT1 is a rate-limiting step for TAG synthesis and CmGPAT1 plays a critical role in TAG synthesis in *C. merolae*. Thus, we decided to further investigate the effects of CmGPAT1 overexpression on TAGs and other lipids to assess the scope for increasing TAG accumulation by manipulation of CmGPAT1 expression.

### Enhanced TAG productivity by CmGPAT1 overexpression

**Figure 4:**
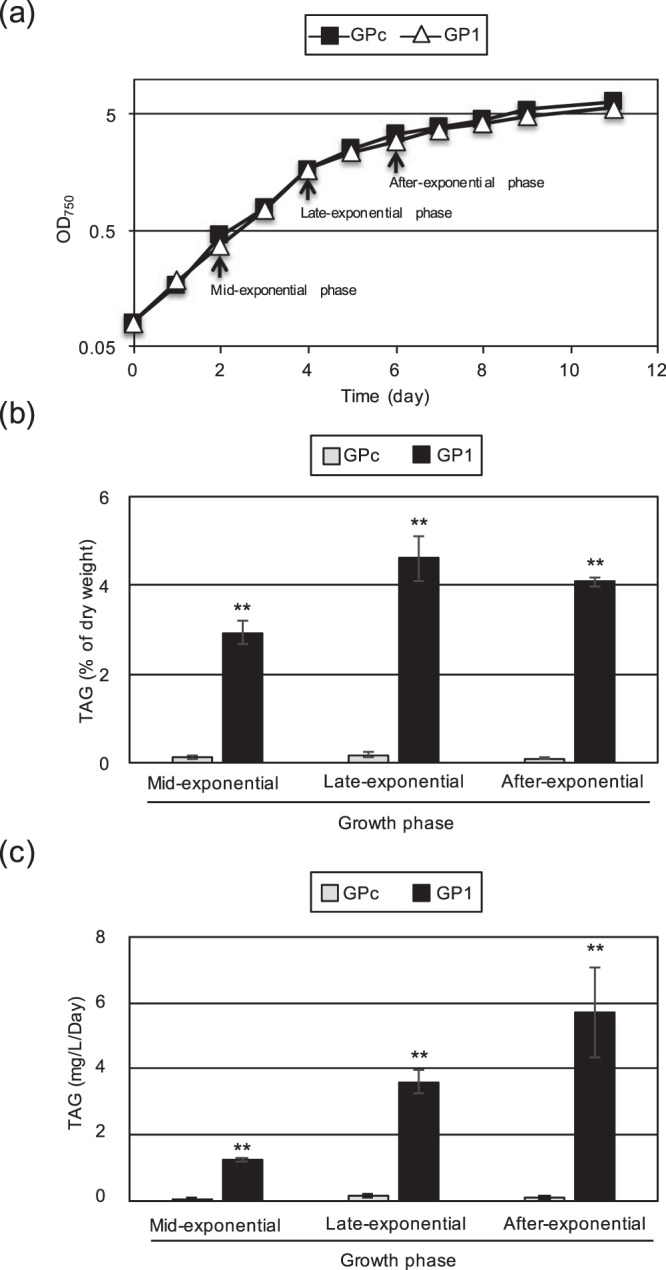
Improved TAG productivity by CmGPAT1 overexpression. (**a**) Sequential growth of the GPc and GP1 strains. The growth of both strains under normal growth conditions was monitored by OD_750_. Arrows indicate the sampling times for the TAG measurements shown in panels b and c. (**b**,**c**) TAG productivity in the GP1 strain. The TAG contents in GPc and GP1 were measured at the mid-exponential, late-exponential, and after-exponential phases, and are shown using two different indexes, percentage of dry weight (**b**) and mg/L/Day (**c**). Other details are the same as in Fig. 3.

In most cases, the growth of microalgae is inhibited under TAG accumulation conditions, such as nitrogen-deletion, phosphorus-depletion, and TOR-inactivation conditions^8,22^. Interestingly, the growth of GP1 was comparable to that of the control strain, GPc, despite the high level of TAG accumulation in GP1 (Fig. 4a). Next, we quantified the TAG amounts in three growth phases: the mid-exponential, late-exponential and after-exponential phases, and calculated the TAG productivity during each phase. The amount of TAGs was the greatest during the late-exponential phase (about 4.6% of dry weight) and was slightly reduced during the after-exponential phase (Fig. 4b). With respect to TAG productivity, as shown in Fig. 4c, the value (mg/L/day) was drastically increased compared with that of GPc, and increased with incubation time to 1.2 (29.9-fold increase compared with the control), 3.6 (24.7-fold), and 5.7 mg/L/Day (56.1-fold) during the mid-exponential, late-exponential and after-exponential phases, respectively. These results clearly indicated that CmGPAT1 overexpression improves TAG productivity, and the step catalyzed by CmGPAT1 is a suitable target for the improvement of TAG production in this alga.

### Contents of phospholipids and glycolipids in the CmGPAT1 overexpression strain and fatty acid distribution in TAGs

**Figure 5:**
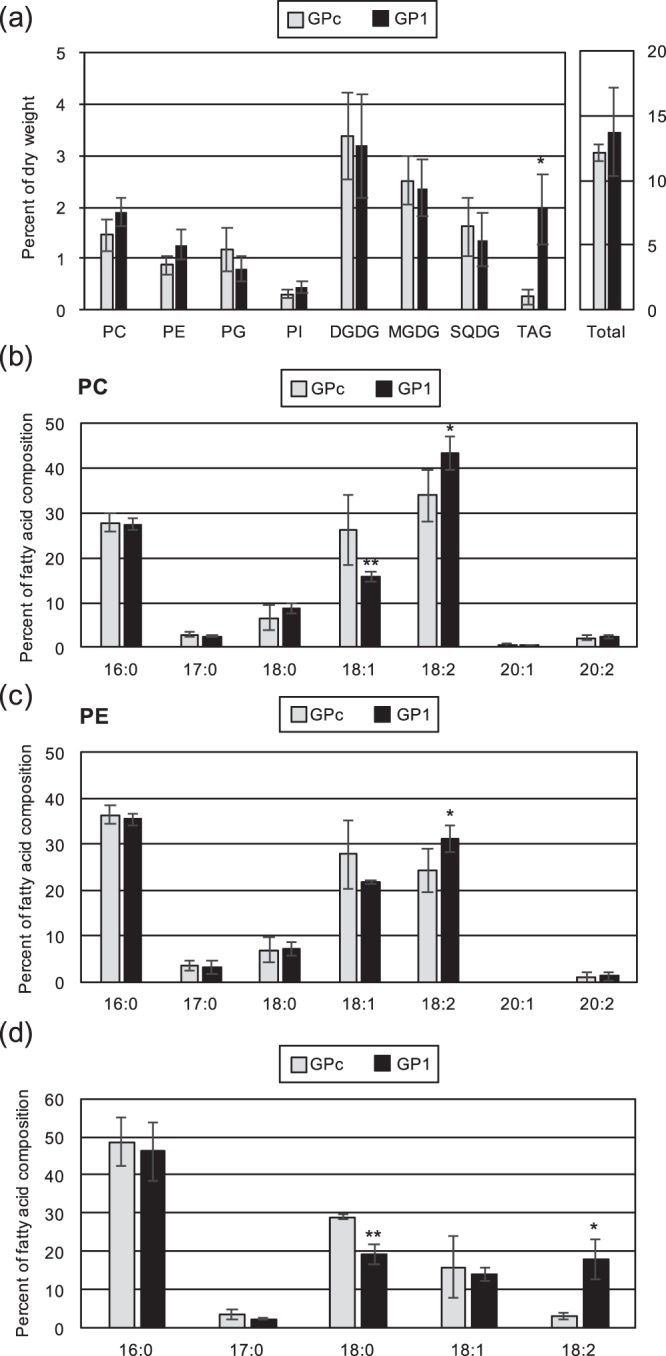
Contents of phospholipids and glycolipids and their fatty acid compositions in the CmGPAT1 overexpression strain. (**a**) Lipid contents in GPc and GP1 cells. Values of indicated lipids are averages of four independent experiments and represent percentages of dry weight. Error bars indicate SD. Other details are the same as in Fig. 3c. (**b**,**c**) Fatty acid composition of the purified PC (**b**) and PE (**c**) in GPc and GP1 cells. Other details are the same as in Fig. 3c. (**d**) Positional distribution of fatty acids in the TAGs. TAGs from GPc and GP1 were purified and subjected to the lipase reaction. After isolation, the released fatty acids were quantified. Other details are the same as in Fig. 3c.

The *de novo* synthesis pathway of TAGs in the ER is partially identical to that of phospholipids, including phosphatidylcholine (PC), phosphatidylethanolamine (PE), phosphatidylglycerol (PG), and phosphatidylinositol (PI). Thus, we also investigated the contents of these phospholipids in GP1. The contents of glycolipids, digalactosyldiacylglycerol (DGDG), monogalactosyldiacylglycerol (MGDG), and sulfoquinovosyldiacylglycerol (SQDG), which are synthesized in the chloroplast, were also measured as controls. In this study, phosphatidic acid (PA) content was not measured because the PA spot on the TLC plate was under the detection limit in our experimental conditions (Fig. S3). As shown in Fig. 5a, the contents of phospholipids and glycolipids were almost the same in the two strains. Additionally, the content of total lipids estimated by the amounts of phospholipids, glycolipids, and TAGs was almost same in the two strains (Fig. 5a). With respect to the fatty acid composition of each lipid, we found different compositions in PC and PE (Figs 5b,c, and S4). In both PC and PE, 18:2 fatty acids were significantly increased in the GP1 strain compared with GPc, and a reduction of 18:1 fatty acids was observed in PC. These results raised the possibility that lysophosphatidic acids (LPAs) containing 18:2 synthesized dependently on CmGPAT1 were used for phospholipid biosynthesis. To examine this, the positional distribution of fatty acids in the TAGs was checked by limited hydrolysis of the acyl ester linkage at the *sn*-1 and *sn*-3 positions with a lipase from *Rhizopus niveus*. If the hypothesis is correct, relative level of 18:2 in the released fatty acids that derived from TAGs in GP1 should increase compared to that in GPc. As shown in Fig. 5d, the relative levels of 18:2 and 18:0 in *sn*-1/*sn*-3 positions were significantly increased and decreased, respectively, suggesting that CmGPAT1 has a substrate preference for 18:2.

### Contents of free fatty acids in the CmGPAT1 overexpression strain

**Figure 6:**
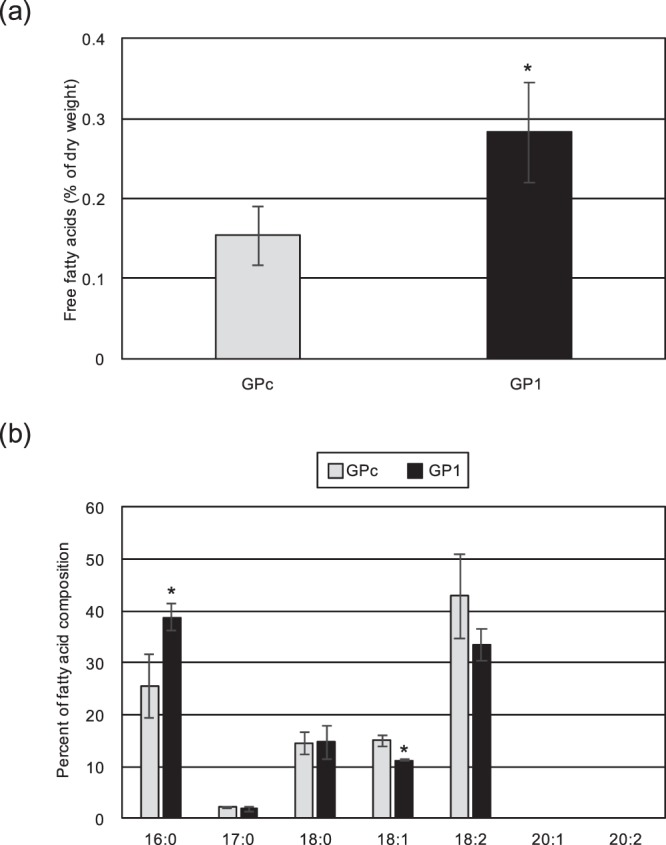
Contents of free fatty acids and the fatty acid composition in the CmGPAT1 overexpression strain. (**a**) Free fatty acid contents in GPc and GP1 cells. Values of indicated lipids are averages of three independent experiments and represent percentages of dry weight. Other details are the same as in Fig. 3c. (**b**) Fatty acid composition of the purified free fatty acids in GPc and GP1 cells. Other details are the same as in Fig. 3c.

GPAT catalyzes the synthesis of LPA from glycerol-3-phosphate and long-chain acyl-CoA as the substrate. Thus, we also measured the amount of free fatty acids, the substrate for acyl-CoA biosynthesis, in GP1. Interestingly, the quantification analysis showed that the amount of free fatty acids was significantly increased to approximately 1.8-fold in GP1 compared with GPc (Fig. 6a). With respect to the species of fatty acids in the isolated free fatty acids, 16:0 and 18:1 fatty acids were significantly increased and decreased, respectively (Fig. 6b).

## Discussion

In this study, we showed that overexpression of the ER-localized CmGPAT1 leads to TAG accumulation under normal growth conditions without any negative impact on cell growth in *C. merolae* (Figs 3 and 4). Consequently, the TAG amount at the late-exponential phase (4.6% of dry weight) considerably exceeded that at 72 h after nitrogen depletion in the wild-type (3.1% of dry weight)^22^, and the TAG productivity was drastically improved (56.1-fold increase). As mentioned in the Introduction, microalgae are considered the most promising next-generation biofuel feedstock. Therefore, the findings in this study are important from a biofuel production standpoint, and provide fundamental knowledge about the regulation of TAG synthesis in microalgae.

Although previous studies using other microalgae have indicated that expression of TAG synthesis-related enzymes is important for TAG accumulation in cells^10^, TAG productivities were drastically increased (more than 56 times) for the first time in algae by ER-localized GPAT overexpression in this study (Figs 3 and 4). Overexpressed CmGPAT2 was also detected in the microsome fraction as with CmGPAT1; however, LD formation and TAG accumulation were not affected (Fig. 3). One explanation for this result is that the activity of CmGPAT2 is regulated at the post-transcriptional level and/or the exogenous protein level is not sufficient. The function of CmGPAT2 and the different roles of CmGPAT1 and CmGPAT2 in lipid synthesis will be the subject of future studies. Notably, overexpression of CmGPAT3, which is localized in the chloroplast^37^, did not lead to LD formation under normal growth conditions (Fig. S5). Previous study using a marine diatom, *Phaeodactylum tricornutum*, indicated that overexpression of chloroplast localized GPAT increased the neutral lipid content only 2-fold^38^. These observations suggest that the reaction catalyzed by chloroplast localized GPAT is not important for TAG accumulation compared with ER-localized GPAT in microalgae.

Among previous studies reporting the improvement of TAG production, the interrelationship between type-2 DGAT and TAG accumulation has been well studied. In *Chlamydomonas reinhardtii*, induction of *DGTT4* using a sulfoquinovosyldiacylglycerol 2 promoter that is activated during phosphorus depletion resulted in increased TAG accumulation compared with the wild-type^8^. TAG biosynthesis was also significantly elevated in *Nannochloropsis oceanica* and *Neochloris oleoabundans* by overexpression of their respective *DGAT2* genes^39,40^. Therefore, the step catalyzed by DGAT is thought to be a rate-limiting step for TAG biosynthesis in these microalgae. However, the regulation of TAG synthesis by DGAT seems to be complex, because three type-2 DGATs, *DGTT1–3*, have been independently overexpressed in *C. reinhardtii* but did not increase intracellular TAG accumulation compared with the wild-type under normal growth conditions or nutrient-depletion conditions^41^. In *C. merolae*, only transcripts of *CMB069C*, which encodes a type-2 DGAT, were increased among the four DGAT genes by TOR-inactivation with rapamycin^22^. We constructed a CMB069C overexpression (CMB069Cox) strain and investigated LD formation in cells grown under normal growth conditions. LD formation was not observed, the same as in CmGPAT2 and CmGPAT3 overexpression strains (Fig. S6). These observations indicate that further investigations are required to determine which reactions are rate-limiting steps and suitable targets for improving TAG productivity in different microalgae. Once again, we emphasize that the reaction catalyzed by CmGPAT1 is a rate-limiting step for TAG production at least in *C. merolae*.

The best suitable character of GP1 strain for TAG production is that the strain shows no growth inhibition even with the accumulation of high levels of TAGs in the cells. One explanation for this character might be the normal lipid composition except for TAGs in GP1 strain. It has been reported that the contents of phospholipids and galactolipids are drastically changed in response to the TAG accumulation conditions, such as nitrogen depletion or phosphorus depletion^8,42^. These lipids are the predominant and indispensable cellular components for cell growth and photosynthesis. The levels of phospholipids seem to be strictly regulated since CmGPAT1 catalyzes the first step of lipid synthesis of phospholipids and TAGs on the ER. Further, the upregulation of fatty acid synthesis might also contribute to the normal growth rate in GP1 strain (see below).

One clear feature of the TAGs in the CmGPAT1 overexpression strain was an increase of 18:2 fatty acids (Fig. 3c). Although more detailed analysis on substrate preference of CmGPAT1 by *in vitro* biochemical assay will be the subject of future study, the analysis of the positional distribution of fatty acids in the TAGs suggested that CmGPAT1 has a substrate preference for 18:2 fatty acids (Fig. 5d). *De novo* TAG/PC/PE and PI/PG are synthesized through diacylglycerol (DAG) and CDP-DAG as intermediates, respectively. The relative fatty acid composition of 18:2 was also increased in PC and PE but not in PI or PG (Figs 5 and S4), suggesting CDP-DAG containing 18:2 is not predominantly used for PI or PG synthesis or for supporting the CmGPAT1 substrate preference. Recently, Toyoshima *et al*.^42^ reported that TAGs accumulated under nitrogen-depletion conditions contain mainly 18:2 at the *sn*-2 position, which could be derived from PC in *C. merolae*. PC is also significantly increased in response to nitrogen depletion and contains this fatty acid at the *sn*-2 position^42^, suggesting an increase of the PC pool and/or Acyl-CoA pool in which the level of 18:2 is high. In the GP1 strain, the PC amount was not changed (Fig. 5a) and the content of 18:2 in the free fatty acids was decreased compared with the control strain (Fig. 6b). Therefore, the TAG accumulation mechanism under nitrogen depletion and in GP1 is likely to be different.

The other remarkable feature in the CmGPAT1 overexpression strain was the increased amount of free fatty acids (Fig. 6a). Notably, the relative content of 16:0 fatty acids in the free fatty acids was significantly increased (Fig. 6a). In *C. merolae*, 16:0-acyl carrier protein (ACP) and 18:0-ACP are synthesized in the chloroplast and are hydrolyzed by thioesterase to release free fatty acids that are exported from the chloroplast^43^, indicating that CmGPAT1 overexpression leads to an increase of *de novo* fatty acid synthesis. Although the reason and mechanism for the increase are unclear at present, intermediates of the *de novo* TAG synthesis pathway could affect the 16:0 level; for example, PA and DAG are well-known second messengers that regulate some signaling pathways^44,45^. The resultant increase of free fatty acids by CmGPAT1 overexpression provides suitable substrates of lysophosphatidic acid acyltransferase (LPAT) and DGAT for TAG synthesis, which would also contribute to the high level of TAG accumulation in the GP1 strain.

The roles of CmGPAT1 and CmGPAT2 in TAG accumulation were investigated using an overexpression approach because both transcripts were increased under both nitrogen depletion and TOR-inactivation by rapamycin^22^. Previous studies have suggested that TOR-signaling is one of the major pathways that regulate TAG accumulation^22,23^, suggesting that TOR regulates TAG accumulation by changing CmGPAT1 expression. TOR is a conserved protein kinase that is indispensable for cell growth among eukaryotes (see Introduction). Furthermore, it has been shown that *A. thaliana* GPAT9, an ortholog of CmGPAT1 (Fig. 1), has been highly conserved throughout evolution with homologs found in most plants, features consistent with essential housekeeping functions^25^. It is therefore expected that the interrelationship between TOR and ER-localized GPAT in the regulation of TAG accumulation is conserved among plant lineages. Revealing the fundamental regulation by TOR and its mechanism under particular physiological conditions would be useful to further improve TAG productivity in the cells. For example, identifying TOR activity-dependent transcription factors that regulate *CmGPAT1* transcription is an attractive subject as the next step. If we can identify such regulators and modify their function, TAG productivity will be further improved because transcription factors affect the expression of a wide range of genes including *CmGPAT1*-related genes. This kind of approach based on the fundamental molecular mechanism of TAG synthesis should lead to successful biofuel production using microalgae.

## Methods

### Strains and growth conditions

*Cyanidioschyzon merolae* 10D wild-type, T1^46^, GPc, GP1, GP2, GP3, and CMB069Cox strains were grown at 40 °C under continuous white light (50 μmol m^−2^ s^−1^) in liquid MA2 medium^13^ at pH 2.5 bubbled with air supplemented with 2% CO_2_. For cultivation of T1, uracil and 5-fluoroorotic acid were added to MA2 medium (0.5 mg/mL final concentration).

### Phylogenetic analysis

A phylogenetic tree based on 303 unambiguously aligned amino acid positions of GPAT proteins and their three homologs in *C. merolae* was constructed as described previously^47^.

### Construction of CmGPAT1, CmGPAT2, CmGPAT3, and CMB069C overexpression strains

The three GPAT (*CMA017C*, *CMK217C*, and *CMJ027C*) and *CMB069C* genes were amplified with the primer sets shown in Table S1 and *C. merolae* genomic DNA as a template. Each PCR-amplified gene was then cloned into *Sma*I-digested pSUGA^48^ using an In-Fusion HD cloning kit to create pSUGA-A017, pSUGA-K217, pSUGA-J027, and pSUGA-B069. The T1 strain was transformed with pSUGA-A017, pSUGA-K217, pSUGA-J027, pSUGA-B069, and pSUGA, as previously described^15^ to obtain the GP1, GP2, GP3, CMB069Cox, and GPc strains, respectively. Each cloned protein was fused with a FLAG-tag and the expression was regulated by the strong *APCC* promoter^48^.

### RNA preparation and quantitative real-time PCR

Total RNA preparation and quantitative real-time PCR (qRT-PCR) were performed as described previously^14^ with few modifications. *C. merolae* cells from 20–30 mL of culture were harvested by centrifugation. The resultant pellet was dissolved in 500 µL of RNA extraction buffer (50 mM Tris-HCl pH 6.8, 5 mM EDTA, 0.5% (w/v) SDS), and then 500 µL of acidic phenol was added. The tube was incubated at 65 °C for 8 min, with intermittent mixing. After centrifugation of the tube, the supernatant was mixed with an equal volume of phenol:chloroform:isoamyl alcohol (25:24:1). After centrifugation, the supernatant was collected in a new tube and precipitated using ethanol. Isolated RNA was treated with DNase I, and further treated with phenol:chloroform:isoamyl alcohol (25:24:1) followed by ethanol precipitation. The resultant purified RNA was used as total RNA for the quantitative real-time PCR (qRT-PCR) analyses in this study. In the step of complementary DNA (cDNA) synthesis, cDNA fragments were synthesized using 0.1–0.5 μg total RNA with a ReverTra Ace^®^ qPCR RT Master Mix with gDNA Remover Kit (TOYOBO), according to the manufacturer’s protocol. Primers used in qRT-PCR analyses are shown in Table S2.

### Immunoblot analysis

Immunoblot analysis was essentially performed as described previously^12,47^ with slight modifications. Mouse anti-FLAG (Sigma), rabbit anti-tubulin^34^, rabbit anti-EF-Tu^35^, rabbit anti-RbcL^36^, and rat anti-calnexin^34^ antibodies were used at a dilution of 1:5000 to detect FLAG-fused protein, tubulin, RbcL and calnexin, respectively. An HRP-conjugated anti-mouse, rabbit or rat IgG antibody (Thermo Fisher Scientific) was used as a secondary antibody at a dilution of 1:5000. The signals were detected using ImmunoStar Zeta (FUJIFILM Wako Pure Chemical Industries) and a chemiluminescent image analyzer Lumino Graph I (WSE-6100H, ATTO CORPORATION).

### Isolation of microsomes

Isolation of microsomes from *C. merolae* was performed as described previously with slight modifications^49^. *C. merolae* logarithmic growth phase cells (OD_750_ = 0.5–0.6, approximately 100 ml) were harvested by centrifugation. The cells were suspended in 4 ml of Microsome Lysis Buffer (20 mM Tris-HCl pH 7.9, 10 mM MgCl_2_·6H_2_O_2_, 1 mM EDTA, 5% (v/v) glycerol, 0.3 M sucrose, 1 mM DTT, Complete Mini protease inhibitor), and broken by sonication (BRANSON SONIFIER 250, Duty Cycle 50, Output Control 1.0, 10 times). After centrifugation (17,500 *g*, 4 °C, 20 min), the supernatant was collected as total protein. The total protein was subjected to ultracentrifugation (105,000 *g*, 4 °C, 90 min), and the resultant supernatant was collected as soluble protein. The pellet was washed with Microsome Lysis Buffer and again collected after ultracentrifugation under the same conditions described above. The final pellet was collected as the microsomal fraction.

### Indirect immuno-fluorescence microscopy analysis

The GPc, GP1, and GP2 strains grown under the normal growth conditions were fixed with 1% (w/v) paraformaldehyde and 10% (v/v) dimethyl sulfoxide in methanol. Each fixed cell was reacted with a mouse anti-DYKDDDDK FLAG antibody (FUJIFILM Wako Pure Chemical Industries) and a rat anti-calnexin antibody^34^ at the same time in 5% (v/v) Blocking one (nacalai tesque) in PBS. The localizations of CmGPAT1/CmGPAT2 and calnexin were detected with Alexa Fluor 488-conjugated goat anti-mouse IgG antibody and Alexa Fluor 555-conjugated goat anti-rat IgG antibody (Thermo Fisher Scientific), respectively. The fluorescence derived from the secondary antibody and chlorophyll was observed by fluorescence microscopy (BX51, Olympus).

### Lipid analysis

Staining and detection of LDs with BODIPY 505/515 (4,4-difluoro-1,3,5,7-tetramethyl-4-bora-3a,4a-diaza-s-indacene) and quantification of TAGs and free fatty acids by gas chromatography were performed as described previously^22,50^. *C. merolae* cells (40–50 ml) were harvested by centrifugation, frozen immediately in liquid nitrogen, and stored at −80 °C until use. After freeze-drying the cells, about 10 mg of them were used for the total lipid extraction in accordance with the method described previously^51^. The lipids were separated by one-dimensional thin-layer chromatography with hexane:diethyl ether:acetic acid (40:10:1, v/v). After isolation of TAGs, fatty acids contained in the TAGs were converted to fatty acid methyl esters by incubating in 5% (v/v) HCl in methanol at 85 °C for 1 h, and then analyzed with gas chromatography (GC-2014, SHIMADZU). Fatty acid methyl esters derived from the TAGs were identified in comparison with retention time of fatty acid methyl ester standards (Supelco 37-component FAME Mix, Sigma-Aldrich). Pentadecanoic acid, which is not detected in *C. merolae* was used as an internal standard for calculation of the concentration of each fatty acid methyl ester based on peak areas. All experiments were performed in triplicate. Phospholipids and galactolipids were separated by two-dimensional thin-layer chromatography. The first dimension was developed with acetone:benzene:methanol:water (8:3:2:1, v/v) to the top of the plate. After drying for 30 min, the second dimension was developed with hexane:diethyl ether:acetic acid (80:30:1, v/v) until the solvent front reached half the height of the plate. After drying again for 30 min, the plate was further developed in the second dimension with chloroform:acetone:methanol:acetic acid:water (10:4:2:3:1, v/v). Lipids were visualized with 0.01% (w/v) primuline in 80% (v/v) acetone under UV light (365 nm) and each lipid was extracted from the thin layer chromatography plate. The fatty acids contained in each lipid were converted to fatty acid methyl esters similar to those in TAGs. After the methanolysis reaction, 1.0 ml of hexane and 0.5 ml of 0.9% (w/v) NaCl were added to purify the fatty acid methyl esters, which were then analyzed with gas chromatography as described above. The positional distribution of fatty acids within the TAGs was analyzed as described previously^42^. Briefly, TAGs isolated by TLC were transferred to a glass tube and incubated for 15 min after adding 3 ml chloroform:methanol (2:1, v/v). Then, 5 ml of 1% KCl (w/v) was added to the tube and the lower phase was collected after centrifugation (1,000 *g*, 3 min, 4 °C). The purified TAGs were suspended in 7 ml phosphate buffer (pH 7.4) and subjected to a lipase reaction (4 mg of lipase from *Rhizopus niveus* purchased from SIGMA, 37 °C, 60 min). Next, 5 ml of hexane was added to the reaction solution and mixed well. After centrifugation (1,000 *g*, 3 min, 4 °C), the upper phase was collected and the purified lipids were suspended in 0.1 ml of chloroform:methanol (2:1, v/v). The lipids were separated by TLC with hexane:diethyl ether:acetic acid (160:40:4, v/v) and quantified as described above for the other lipids.

## Electronic supplementary material

Supplementary Information
