# Supplementary material for: Accelerated triacylglycerol production without growth inhibition by overexpression of a glycerol-3-phosphate acyltransferase in the unicellular red alga Cyanidioschyzon merolae
Source: Sci Rep. 2018 Aug 17;8:12410. doi: 10.1038/s41598-018-30809-8 (PMC6098107; doi:10.1038/s41598-018-30809-8)
Supplement: Supplementary file 1 — Supplementary Information [file 41598_2018_30809_MOESM1_ESM.pdf]

Accelerated triacylglycerol production without growth inhibition by overexpression of a glycerol-3-phosphate acyltransferase in the unicellular red alga *Cyanidioschyzon merolae*

Satoshi Fukuda[1,†], Eri Hirasawa[2,†], Tokiaki Takemura[1], Sota Takahashi[2], Kaumeel Chokshi[3], Imran Pancha[3], Kan Tanaka[3], and Sousuke Imamura[3,*]

Supplementary Information

Figure S1. Predicted TAG synthesis pathway in *C. merolae*.

GPAT, LPAT, PAP, and DGAT indicate glycerol-3-phosphate acyltransferase, lysophosphatidate acyltransferase, phosphatidic acid phosphatase, and diacylglycerol acyltransferase, respectively. Each enzyme in *C. merolae* is indicated at the right sides of each step. G3P, LPA, PA, DAG, and TAG indicate glycerol-3-phosphate, lysophosphatidic acid, phosphatidic acid, diacylglycerol, and triacylglycerol, respectively.

Figure S2. Total lipid contents of the CmGPAT1 and CmGPAT2 overexpression strains.

Total lipid contents (based on the weight of the extracted total lipids) of GPc, GP1, and GP2 cells grown under the same growth conditions as in Figure 3 were measured and shown as percentages of dry cell weight. Each value represents the mean ± standard deviation (SD) of three independent experiments.

Figure S3. 2D-TLC chromatography of total lipids isolated from *C. merolae*.

PC, PE, PG, PI, DGDG, MGDG, SQDG, and TAG indicate phosphatidylcholine, phosphatidylethanolamine, phosphatidylglycerol, phosphatidylinositol, digalactosyldiacylglycerol, monogalactosyldiacylglycerol, and sulfoquinovosyldiacylglycerol, respectively.

Figure S4. Fatty acid composition of the purified PI, PG, DGDG, MGDG, SQDG, and total lipids in GPc and GP1 cells.

Fatty acid components of PI (a), PG (b), DGDG (c), MGDG (d), SQDG (e), and total lipids (f) are indicated as percentages of the fatty acid composition. Other details are the same as in Figure 3.

Figure S5. Overexpression of CmGPAT3 and its effect on lipid droplet formation.

(a) Transcript level of the *C. merolae GPAT3* gene. The level of *CmGPAT3* transcript in the GP3 strain was analyzed by quantitative real-time PCR and is presented as a relative value (mean of $n = 3 \pm$ S.D.; the value for the control strain GPc is normalized to 1.0).

(b) Expression of FLAG-fused CmGPAT3. Aliquots of total protein (6 µg each)

isolated from the indicated strains were separated by 10% sodium dodecyl sulfate-polyacrylamide gel electrophoresis (SDS-PAGE) and analyzed by immunoblotting with a monoclonal anti-FLAG antibody. Molecular size marker positions are indicated in kDa on the left. The arrowhead indicates the expected position of the FLAG-tagged CmGPAT3 protein. After antibody detection of the signal, the membrane was stained with Coomassie Brilliant Blue, which was used as a loading control (lower panel). (c) BODIPY staining of GPc and GP3 cells. DIC (differential interference contrast microscope) (top) and BODIPY staining (bottom) images are indicated. Each BODIPY staining image was merged with the relevant chlorophyll fluorescence image. *Bar*, 2 μm.

Figure S6. Overexpression of CMB069C and its effect on lipid droplet formation. (a) Transcript level of the *C. merolae CMB069C* gene. The level of *CMB069C* transcript in the CMB069C overexpression (CMB069Cox) strain was analyzed the same way as in Figure S5a. (b) Expression of FLAG-fused CMB069C. FLAG-fused CMB069C protein was detected the same way as in Figure S5b. (c) BODIPY staining of GPc and CMB069Cox cells. DIC (top) and BODIPY staining (bottom) images are indicated. Others are the same as in Figure S5c.

Table S1 Primers used for construction of overexpression strains.

Gene	Primer	Sequence (5′-3′)
CmGPAT1	A017_pSUGA_F	CGTTCGTTGACCCCCATGGCAGCGACCACCGCC
(*CMA017C*)	A017_pSUGA_R	GTCGACTCTAGACCCGAAGCGAGCCGTCTTGGG
CmGPAT2	K217_pSUGA_F	CGTTCGTTGACCCCCATGCTTTTTGTACGCAACTG
(*CMK217C*)	K217_pSUGA_R	GTCGACTCTAGACCCGCGCCAAATGCGCCGGCA
CmGPAT3	J027_pSUGA_F	TTCTTCGTTCGTTGACCCCCATGTGGGTGTCTTGTATTTC
(*CMJ027C*)	J027_pSUGA_R	TGCAGGTCGACTCTAGACCCTTGCGCATCGTACGCACCCA
CMB069C	B069_pSUGA_F	CGTTCGTTGACCCCCATGGCTTTTTGCGATTTGAG
	B069_pSUGA_R	GTCGACTCTAGACCCGTCCAGCAGCAAATGCGC

Table S2 Primers used for qRT-PCR analysis.

Gene	Primer	Sequence (5′-3′)
CmGPAT1	A017_QRT_F1	AAGCACGTCCTCGAGTGTCT
(*CMA017C*)	A017_QRT_R1	TCGGCTTCTGAAGGTGTTCT
CmGPAT2	K217_QRT_F1	AATGCTGGCATTCCTTGTTC
(*CMK217C*)	K217_QRT_R1	CCTCTATGTCCGCTTTGCAT
CmGPAT3	J027_QRT_F1	GGAAATGGAACGTCTCTTCG
(*CMJ027C*)	J027_QRT_R1	CAAAAGATGCACAGGTGACG
CMB069C	B069_QRT_F1	TTTGGGGCTCATCGATTTAC
	B069_QRT_R1	CTGCTGCATCGCGAATATAA

Figure S1

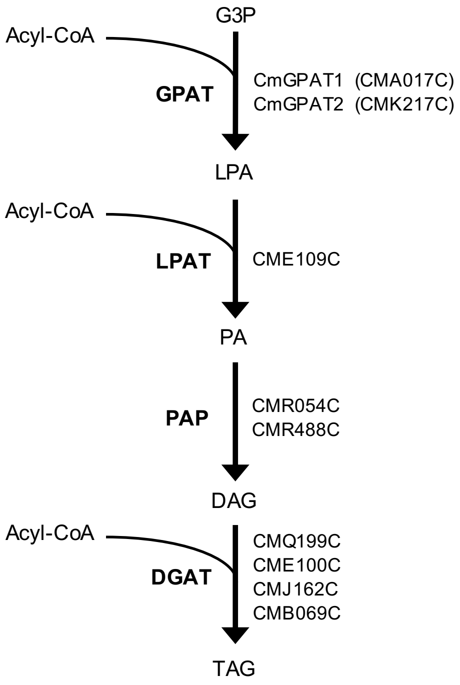
G3P
Acyl-CoA
GPAT
CmGPAT1 (CMA017C)
CmGPAT2 (CMK217C)
LPA
Acyl-CoA
LPAT
CME109C
PA
PAP
CMR054C
CMR488C
DAG
Acyl-CoA
DGAT
CMQ199C
CME100C
CMJ162C
CMB069C
TAG

Figure S2

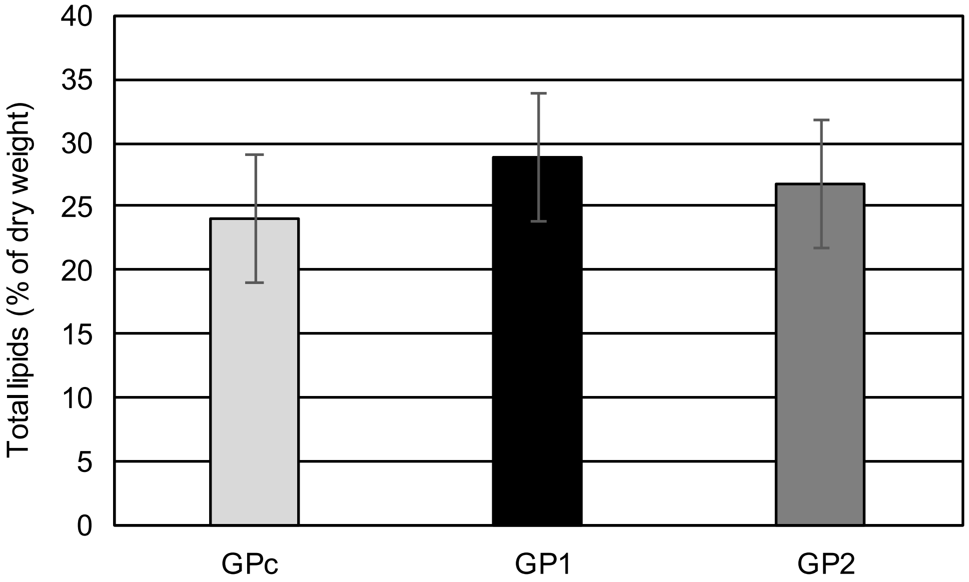

Figure S3

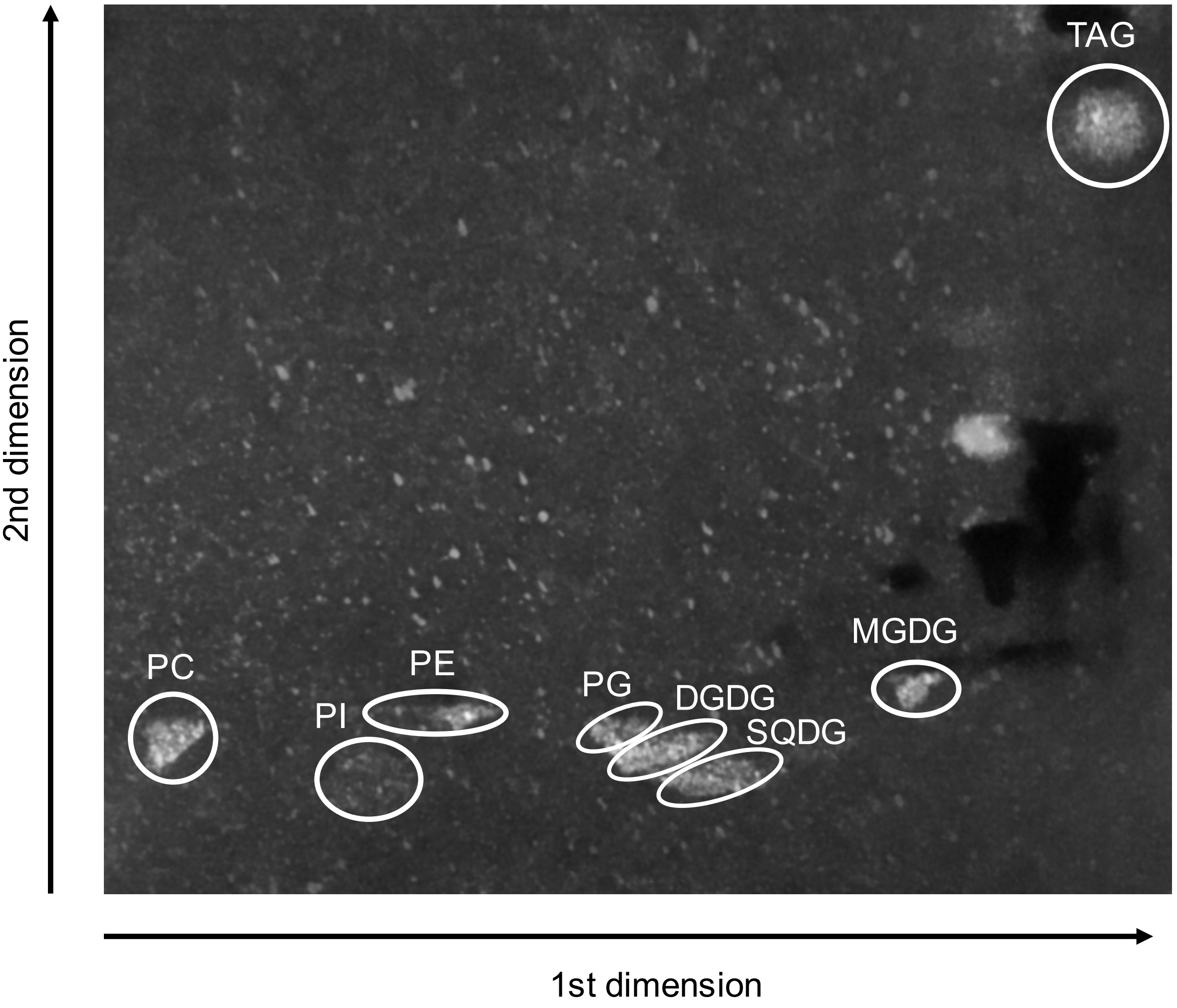

Figure S4

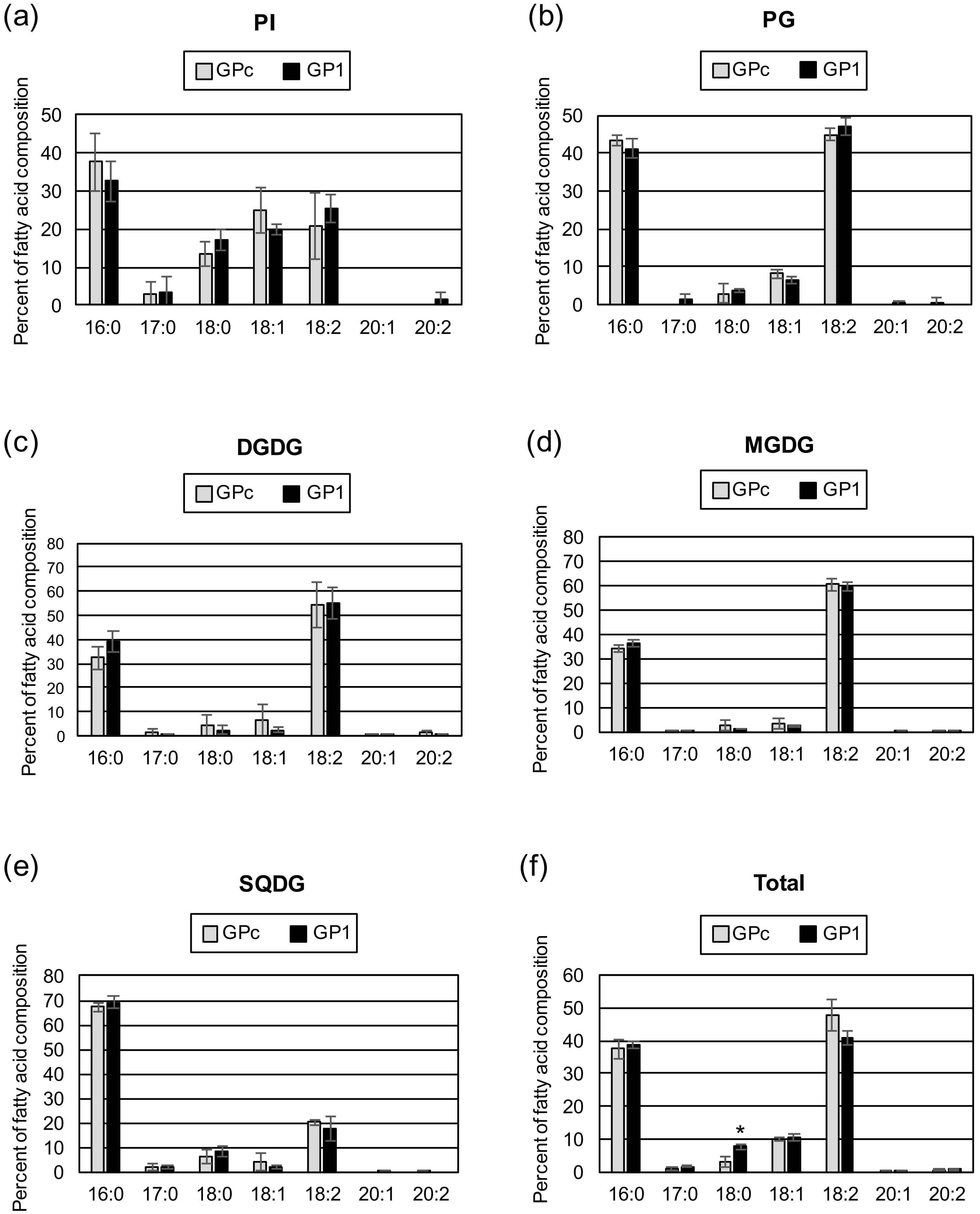

(a)
PI
GPc
GP1
Percent of fatty acid composition
0
10
20
30
40
50
16:0
17:0
18:0
18:1
18:2
20:1
20:2
(b)
PG
GPc
GP1
Percent of fatty acid composition
(c)
DGDG
GPc
GP1
Percent of fatty acid composition
0
10
20
30
40
50
60
70
80
(d)
MGDG
GPc
GP1
Percent of fatty acid composition
(e)
SQDG
GPc
GP1
Percent of fatty acid composition
(f)
Total
GPc
GP1
Percent of fatty acid composition
*

Figure S5

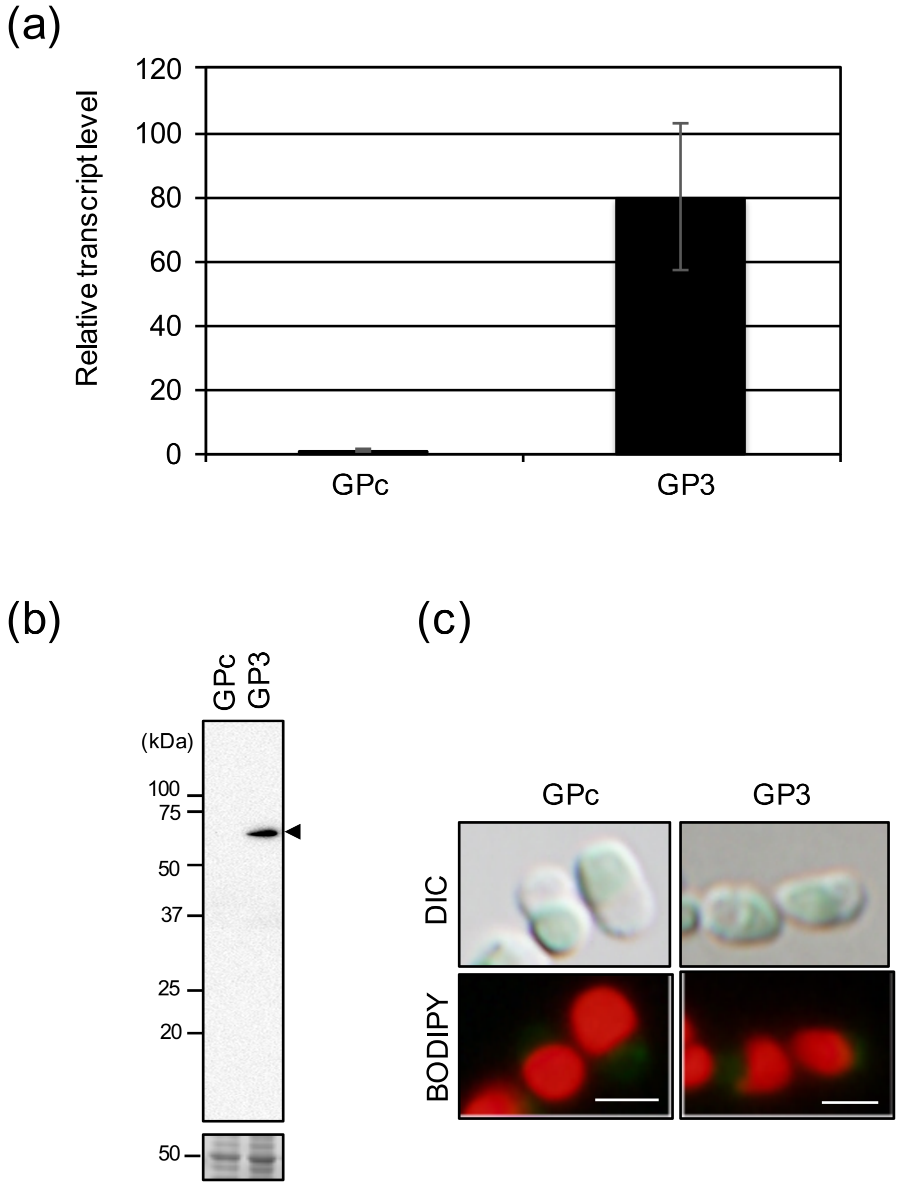

(a)
Relative transcript level
120
100
80
60
40
20
0
GPc
GP3
(b)
GPc
GP3
(kDa)
100
75
50
37
25
20
50
(c)
GPc
GP3
DIC
BODIPY

Figure S6

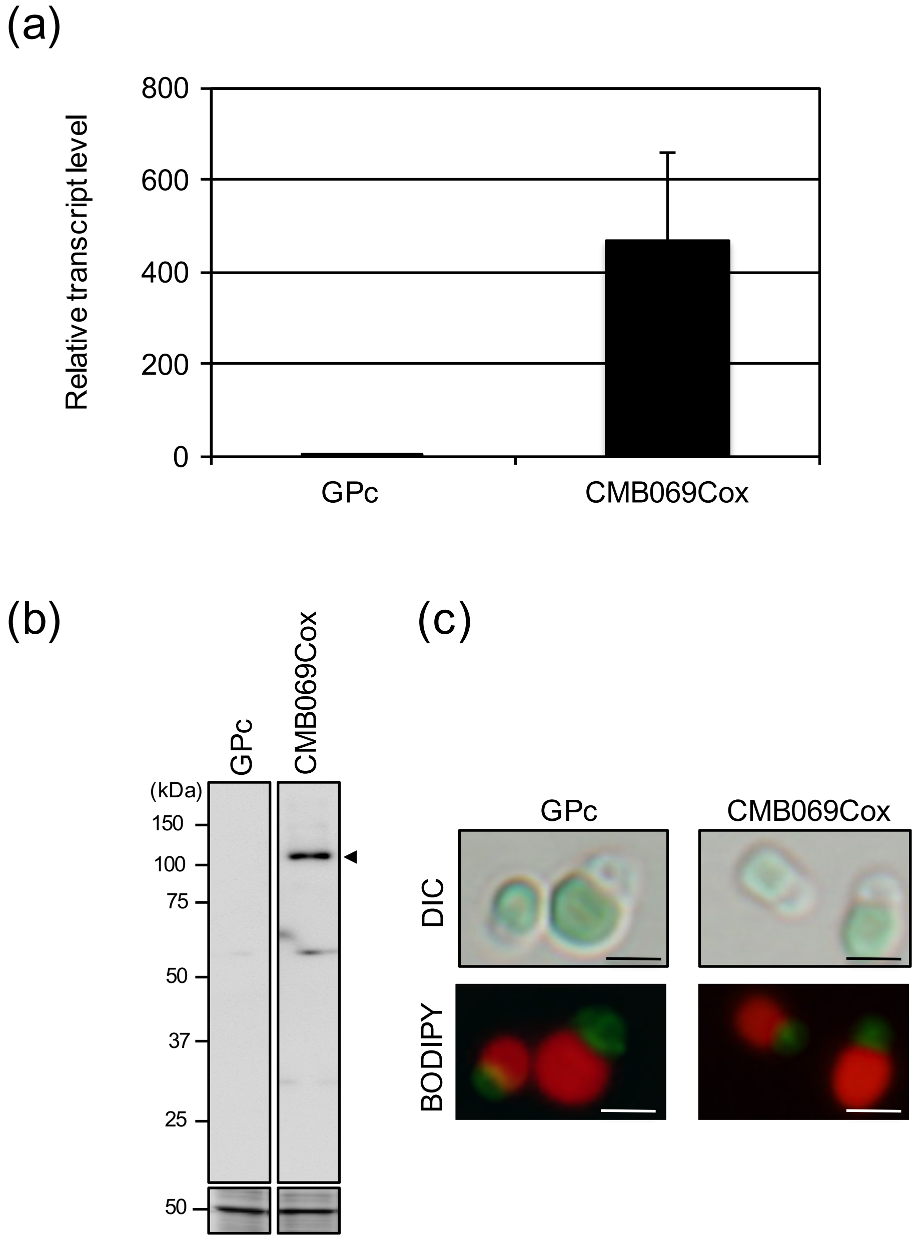
(a)
Relative transcript level
800
600
400
200
0
GPc
CMB069Cox
(b)
GPc
CMB069Cox
(kDa)
150
100
75
50
37
25
50
(c)
GPc
CMB069Cox
DIC
BODIPY
